# Supplementary material for: Examining Health Disparities and Severity of Depression among Sexual Minorites in a National Population Sample
Source: Diseases. 2022 Oct 9;10(4):86. doi: 10.3390/diseases10040086 (PMC9624332; doi:10.3390/diseases10040086)
Supplement: Supplementary file 1 [file diseases-10-00086-s001.zip › diseases-1905741-supplementary.pdf]

Table S1. Association of PHQ-9 Items measuring severity of depression among sexual minorities compared to heterosexuals

	Heterosexual		GLB		
**PHQ-9 Questionnaire Items**	**None to Moderate Depression (n, %)**	**Moderately Severe to Severe Depression (n, %)**	**None to Moderate Depression (n, %)**	**Moderately Severe to Severe Depression (n, %)**	**p-value**
1. Little interest in doing things	6760 (91.4)	637 (8.6)	367 (86.2)	59 (13.8)	0.002
2. Feeling down, depressed, or hopeless	6887 (93.1)	510 (6.9)	362 (85.0)	64 (15.0)	<0.001
3. Trouble sleeping or sleeping too much	6274 (84.8)	1125 (15.2)	344 (80.8)	82 (19.2)	0.032
4. Feeling tired or having little energy	6197 (83.7)	1203 (16.3)	329 (77.2)	97 (22.8)	0.008
5. Poor appetite or overeating	6677 (90.2)	722 (9.8)	355 (83.3)	71 (16.7)	0.003
6. Feeling bad about yourself	6971 (94.2)	428 (5.8)	364 (85.4)	62 (14.6)	<0.001
7. Trouble concentrating on things	6950 (93.9)	450 6.1)	371 (87.1)	55 (12.9)	<0.001
8. Moving or speaking slowly or too fast	7142 (96.5)	257 (3.5)	398 (93.4)	28 (6.6)	<0.001
9. Thought you would be better off dead	7323 (99.0)	73 (1.0)	415 (97.4)	11 (2.6)	0.011

PHQ-9: Patient Health Questionnaire – 9; GLB: Gay/Lesbian/Bisexual; OR: Odds Ratio; CI=Confidence Interval; Boldface p-value indicates statistical significance at <0.05 on Chi-squared analysis

Table S2. Odds Ratio for behavior characteristics among sexual minorities compared to heterosexual**.**

**Variables**	**OR (95%CI)**	**p-value**
Seen mental health professional past year	2.36 (1.65, 3.38)	<0.000
Ever told by doctor, you had genital warts	1.05 (0.49, 2.25)	0.893
Ever told by doctor, you had chlamydia	2.27 (0.96, 5.37)	0.061
Ever told by doctor, you had gonorrhea	1.07 (o.24, 4.74)	0.925
Ever told by doctor, you had genital herpes	1.85 (1.12, 3.04)	0.017
Ever used cocaine/heroin/methamphetamine	1.83 (1.29, 2.58)	0.001
Ever used a needle to inject illegal drug	1.20 (0.63, 2.25)	0.571
Do you now smoke cigarettes?	1.31 (0.93, 1.83)	0.115
Ever have 4/5 or more alcoholic drinks every day?	1.20 (0.82, 1.76)	0.333

OR: Odds Ratio; CI=Confidence Interval; Boldface p-value indicates statistical significance at <0.05
